# Supplementary material for: A Far-Red Fluorescent Probe to Visualize Gram-Positive Bacteria in Patient Samples
Source: ACS Infect Dis. 2024 Apr 17;10(5):1545–51. doi: 10.1021/acsinfecdis.4c00060 (PMC11091877; doi:10.1021/acsinfecdis.4c00060)
Supplement: Supplementary file 1 — id4c00060_si_001.pdf [file id4c00060_si_001.pdf]

Supporting Information for

A Far-Red Fluorescent Probe to Visualize Gram-Positive Bacteria in Patient Samples

Krittapas Jantarug,[1,7] Vishwachi Tripathi,[2,7] Benedict Morin,[3] Aya Iizuka,[3] Richard Kuehl,[3,4,6] Mario Morgenstern,[6] Martin Clauss,[6] Nina Khanna,[3,4,5] Dirk Bumann,[2] and Pablo Rivera-Fuentes[1*]

1. Department of Chemistry, University of Zurich, Zurich 8057, Switzerland.
2. Biozentrum, University of Basel, Basel 4056, Switzerland.
3. Department of Biomedicine, University of Basel, Basel 4031, Switzerland.
4. Division of Infectious Diseases and Hospital Epidemiology, University Hospital Basel, Basel 4031, Switzerland.
5. Department of Clinical Research, University Hospital of Basel 4031, Basel, Switzerland.
6. Center for Musculoskeletal Infections, Department of Orthopaedic and Trauma Surgery, University Hospital Basel, Basel 4031, Switzerland.
7. Equal contribution.

*Correspondence to: pablo.riverafuentes@uzh.ch

Contents

1 GENERAL REMARKS 2

2 MATERIALS AND METHODS 2

3 SUPPLEMENTARY FIGURES 4

4 SYNTHETIC PROCEDURES 10

1 General remarks

All reagents and solvents were purchased from commercial sources and used as received. NMR spectra were acquired on Bruker AVANCE II-400 and Bruker AVANCE II 500 instruments. ^{1}H NMR chemical shifts are reported in ppm relative to $SiMe_4$ (δ = 0) and were referenced internally with respect to residual protons in the solvent (δ = 1.94 for acetonitrile and δ = 3.31 for methanol). Coupling constants are reported in Hz. High-resolution mass spectrometry (HRMS) was performed by the MS facility of EPFL and UZH. Reaction progress was followed by ultra-high performance liquid chromatography–mass spectrometry (UHPLC-MS) on a Shimadzu LC-MS 2020 system using electrospray ionization (ESI) and Low-resolution mass spectra (LRMS) were acquired on a Waters spectrometer by using electrospray ionization (ESI). Purification by prep-HPLC was performed using a Büchi Pure-Chromatography-System and Büchi FlashPure columns. IUPAC names of all compounds are provided and were determined using CS ChemDraw 20.1.

2 Materials and methods

Optical spectroscopic methods

Stock solutions were prepared in DMSO (spectrophotometric grade > 99.9%) at concentrations of 5 mM and stored at -80°C. Spectroscopic measurements were conducted in phosphate-buffered saline (PBS). UV-Vis spectra were acquired using a Multiskan SkyHigh Microplate Spectrophotometer (ThermoFisher Scientific) and quartz cuvettes from Thorlabs (10 mm path length) or 96-well plates (Corning). Measurements were carried out as stated at 25°C unless stated otherwise. Fluorescence spectra were acquired using a FS5 Spectrofluorometer (Edinburgh Instruments) equipped with a SC-25 cuvette holder or SC-40 plate reader and quartz cuvettes from Thorlabs (10 mm path length) or 96-well plates (Corning). All measurements were carried out as three technical replicates. The obtained UV-Vis spectra were background corrected. Prism 8.0 was used to process the data and plot the spectra.

Bacterial culture

Bacteria, *S. epidermidis* (ATCC 12228) was grown in TSB in 5 mL culture tubes at 37°C, shaking (180 rpm). Clinical isolated *S. aureus* (PROSA28), lab strain MSSA (ATCC 29213), and lab strain MRSA (ATCC 43300) were grown in 5 mL cation-adjusted MHB in 50 mL culture tubes at 37°C, shaking (180 rpm). Overnight cultures were streaked on TSB agar plates at 37°C for 16-18 hours.

A single colony was picked and inoculated in TSB/cation-adjusted MHB in 5 mL culture tubes at 37°C, shaking (180 rpm) for 16-18 hours.

Minimal Inhibitory Concentration (MIC)

Clinical isolated *S. aureus* (PROSA28), lab strain MSSA (ATCC 29213), and lab strain MRSA (ATCC 43300) were grown in 5 mL cation-adjusted MHB in 50 mL culture tubes at 37°C with shaking (180 rpm). Overnight cultures were streaked on TSB agar plates at 37°C for 16-18 h. A single colony was picked and inoculated in TSB/cation-adjusted MHB in 5 mL culture tubes at 37°C, shaking (180 rpm) for 16-18 h. The bacteria were regrown in cation-adjusted MHB to an OD_{600} = 0.08-0.1. A 96-well plate was loaded with different concentrations of antibiotic and probe. Bacteria (100 µL) were added into 96-well plates and incubated at 37°C for 16-18 h. The variability of bacteria was measured from OD_{600} by a microplate reader.

Live-cell microscopy

All incubations were done at 37°C and cultures were shaken at 180 rpm. We transformed PROSA with a pRN11-derivative[1] carrying a transcriptional fusion of the $P_{pdhABCD}$ promoter and gfp-mut3.1 obtained from plasmid pC183-S3[2] resulting strain called PROSA28-pVT13. PROSA28-pVT13 was streaked on agar containing brain-heart infusion (BHI) (Remel, Cat No. 452472) with 10 mg L^{-1} chloramphenicol. Overnight cultures were prepared by picking a single colony and inoculating BHI containing 10 mg L^{-1} chloramphenicol. Overnight cultures were diluted 1:1000 and grown for 2 hours. OD_{600} was adjusted to 0.003 in BHI and loaded in a CellAsic plate (Millipore, Cat No. B04A-03). Bacteria were flushed with BHI + 0.17 µM unlabeled vancomycin + 0.13 µM Van-JF_{669} for 150 minutes at 10 kPa. Images were taken every two minutes using a Nikon Ti2 widefield microscope with 100X objective (Laser powers 5% in ch640 and 20% in ch488, exposure times 200 ms)

Super-resolution microscopy

A µ-slide, 8-well, glass-bottom plate (Ibidi, Cat No. 80827) was cleaned with UV-generated ozone for 10 minutes and held for 5 minutes at 25 °C. A *S. epidermidis* suspension in LB broth (OD_{600}=0.1) was added to the well and incubated at 25 °C for 10 minutes. After washing three times of milliQ water, 0.1 nM Van-JF_{669} in water was added to the well. Images were taken using a combined Nikon W1 spinning disc (confocal) and N-STORM (SMLM) system equipped with a Nikon 1.49 NA 100x TIRF Apo Plan SR objective. After optimization of the HILO angle, the acquisition of frames was started, using a 638 nm laser at 90 mW power, with an exposure time of 30 ms and a constant frame rate (15.5 Hz). The acquisition was continued until 5000 frames were required. Images were processed by the Picasso software package (https://github.com/jungmannlab/picasso).[3]

Photostability

Photobleaching experiments were performed using a FS5 Spectrofluorometer equipped with an SC-25 cuvette holder. Compounds were measured at a 50 nM concentration and the excitation slit width was adjusted individually for each dye to achieve a power of 2.0 mW (503 nm, 14 nm; 560 nm, 16.5 nm; 590 nm, 20 nm; and 669 nm, 29.9 nm). The emission slit width was set to 0.75 nm and the fluorescence was measured every second for 10 min while keeping the shutter always open. The power was measured at the cuvette holder using a PMD100D compact power and energy meter console equipped with a S120VC standard photodiode power sensor (UV-Extended Si, 200–1,100 nm, 50 mW, Thorlabs GmbH).[4]

3 Supplementary figures

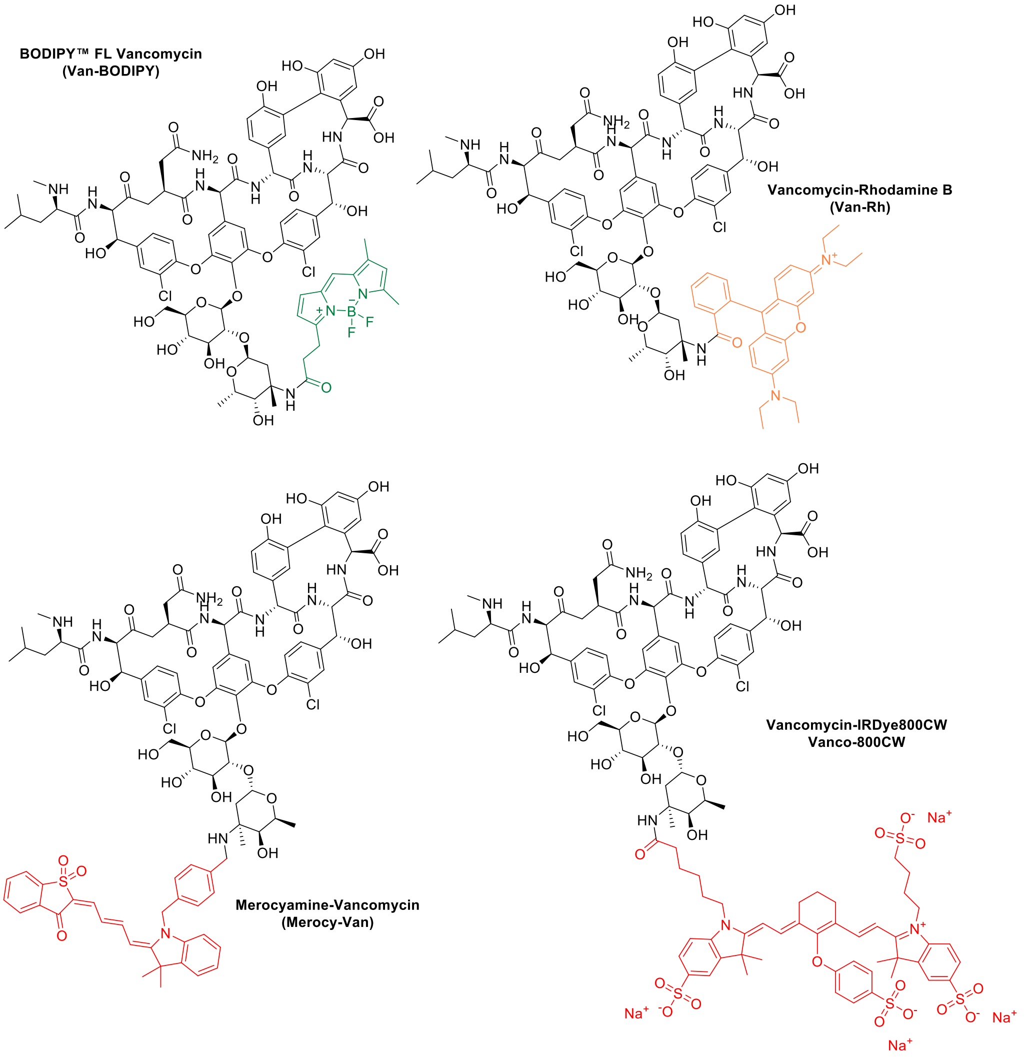

Figure S1. Chemical structures of Van-BODIPY, Van-Rh, Merocy-Van and Vanco-800CW.

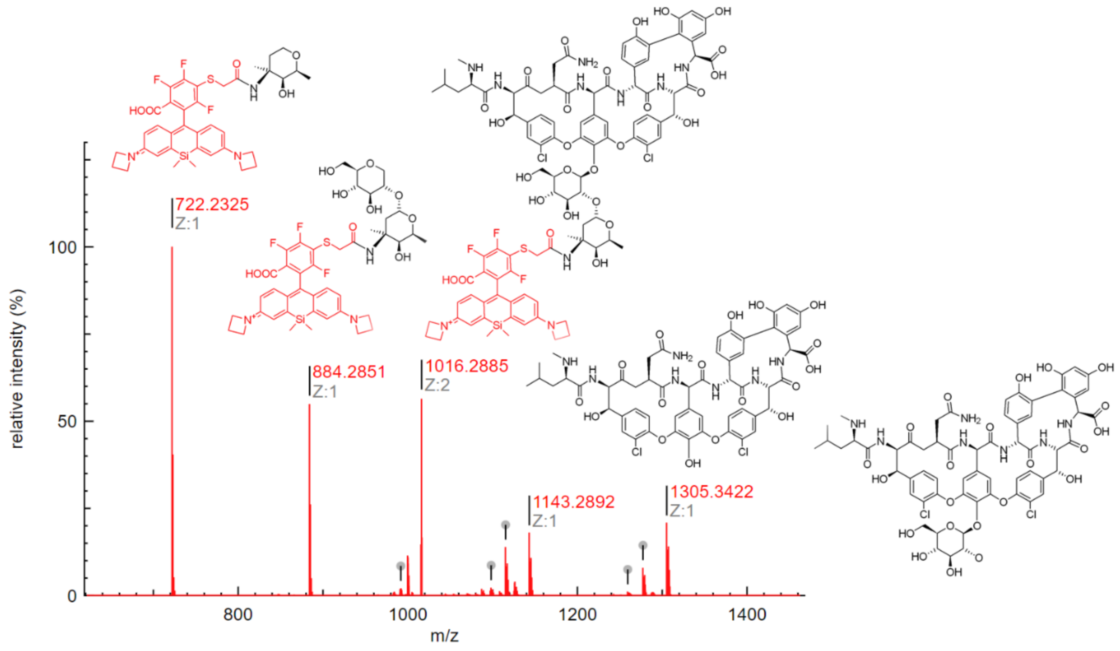

Figure S2. Structure determination of Van-JF$_{669}$. The fragments detected by HRMS correspond to JF$_{669}$ attached to the sugar moiety of vancomycin.

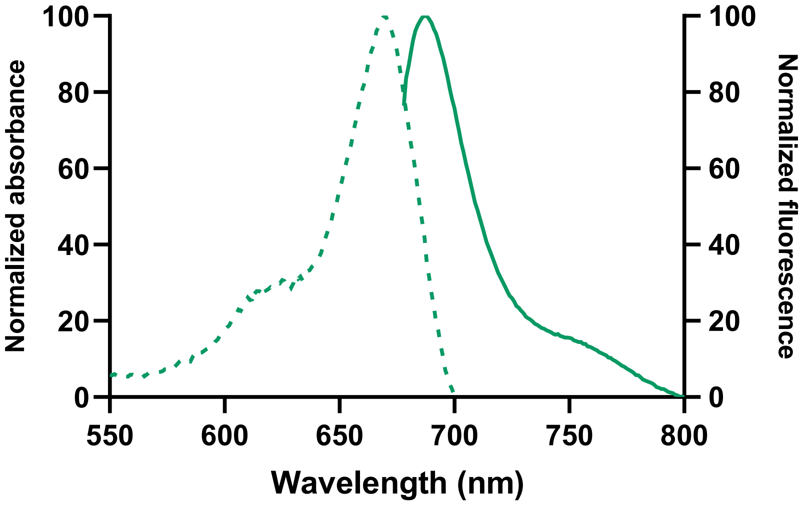

Figure S3. Excitation (---) and emission (—) spectra of Van-JF$_{669}$

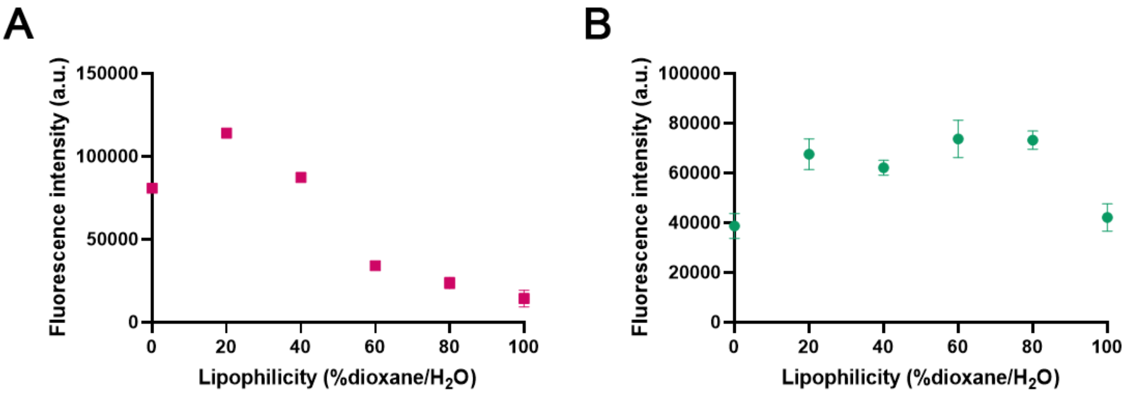

Figure S4. Fluorescence of vancomycin conjugated to different fluorophores. (A) Lipophilicity-dependent changes of Van-JF_{669} fluorescence and (B) lipophilicity-independent changes of Van-BODIPY fluorescence. Symbols indicate mean and whiskers the standard deviation from three technical replicates.

Table S1. Photophysical properties in aqueous buffer of fluorophores used to produce vancomycin probes.

Fluorophore	Quantum yield	Extinction coefficient (M^{-1} cm^{-1})
BODIPY Fl	0.36	80,000
Rhodamine B	0.10	92,200
Merocyanine	0.02	29,500
JF_{669}	0.37	112,000[5]

Measured in HEPES buffer.

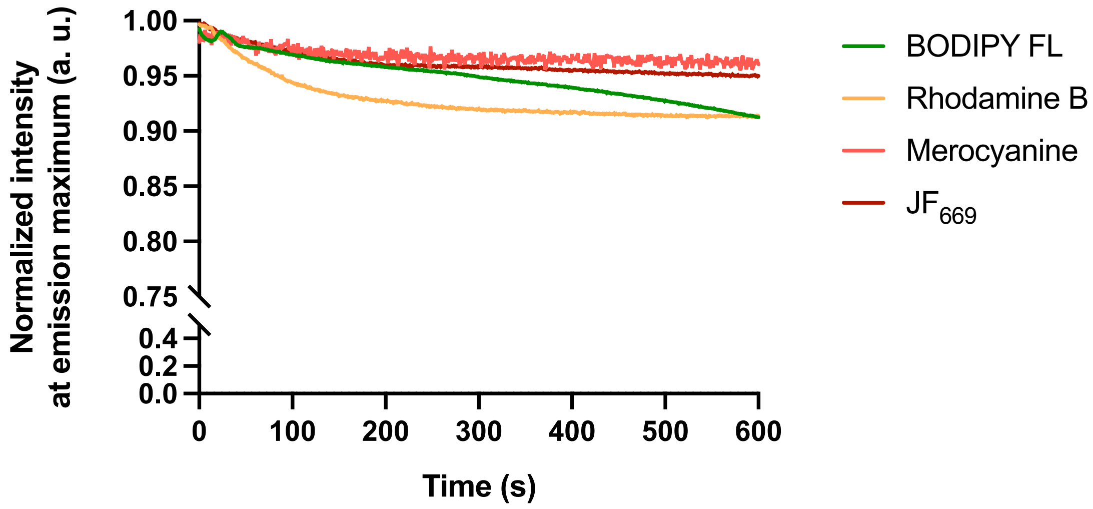

Figure S5. Photobleaching curves measured at a concentration of 50 nM in PBS following irradiation at the maximum excitation wavelength with monochromatic light (2.0 mW power).

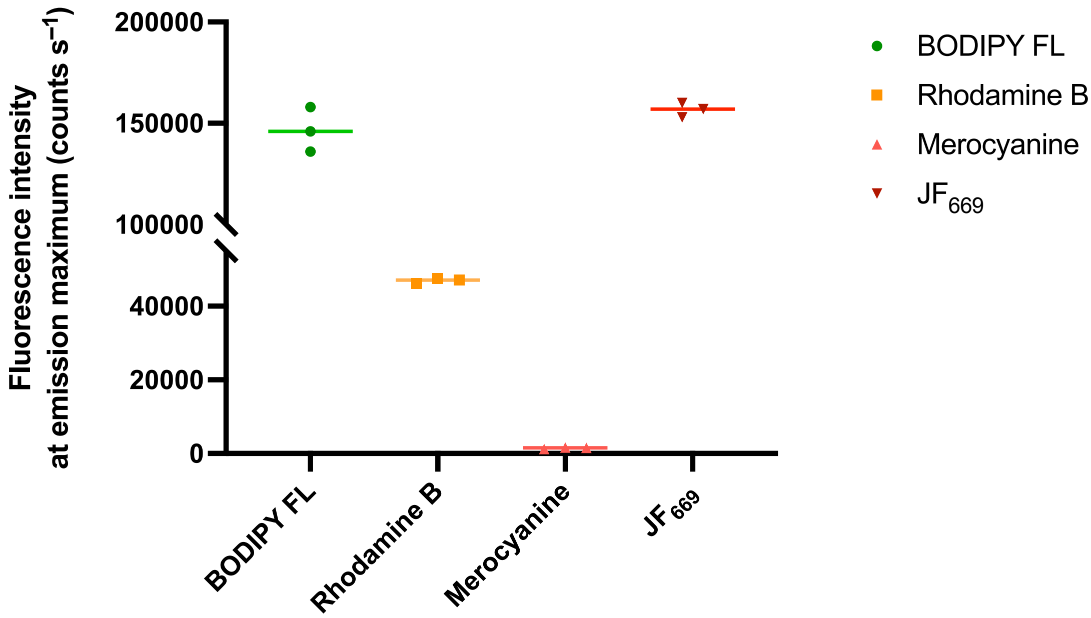

Figure S6. Initial fluorescence intensity (prior to any photobleaching) of 50 nM fluorescent compounds in PBS upon irradiation at the maximum excitation wavelength with monochromatic light (2.0 mW power).

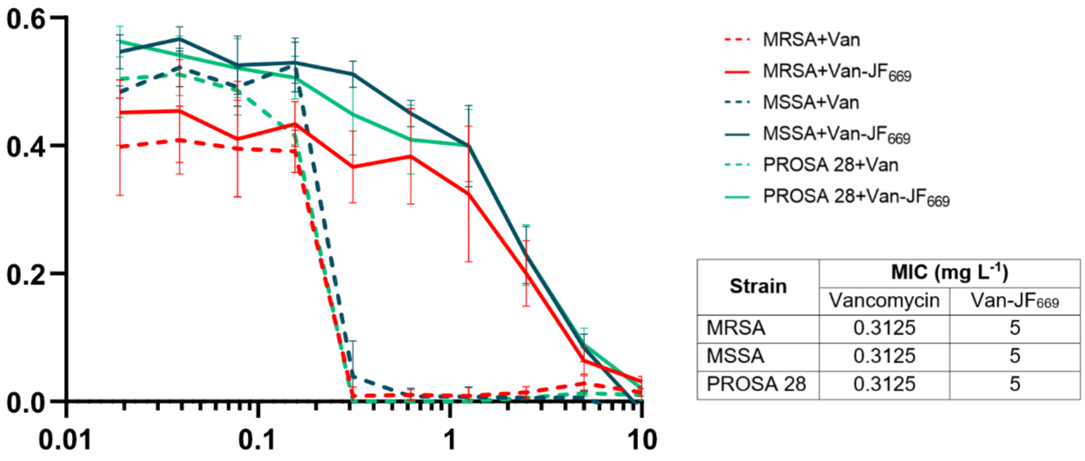

Strain	MIC (mg L^{-1})	
	Vancomycin	Van-JF$_{669}$
MRSA	0.3125	5
MSSA	0.3125	5
PROSA 28	0.3125	5

Figure S7. The minimum inhibitory concentration of vancomycin(van) and Van-JF$_{669}$ in methicillin-resistant *S. aureus* (MRSA, ATCC43300), methicillin-sensitive *S. aureus* (MSSA, ATCC 29213), and clinical isolate *S. aureus* (PROSA28, methicillin sensitive). Lines indicate mean and whiskers standard deviation from four biological replicates.

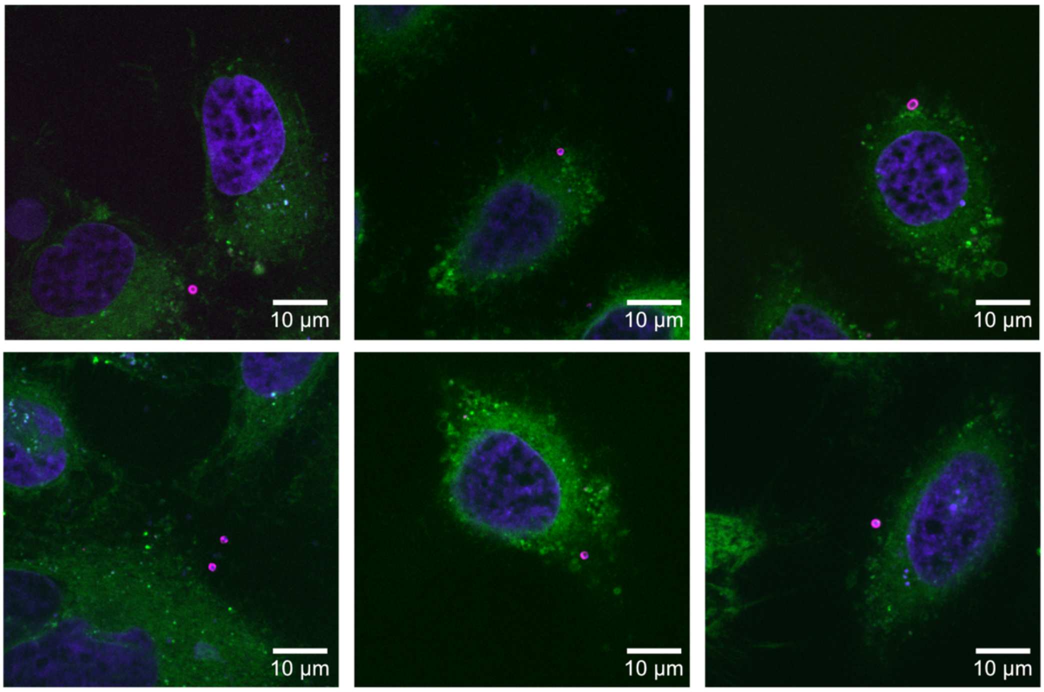

Figure S8. Further examples of micrographs showing selective labeling of *S. epidermidis* co-cultured with HeLa cells and treated with 1.5 µM Hoechst (nuclei = blue), 2 µM ER-Tracker™ Green (endoplasmic reticulum = green) and 10 nM Van-JF_{669} (*S. epidermidis* = magenta).

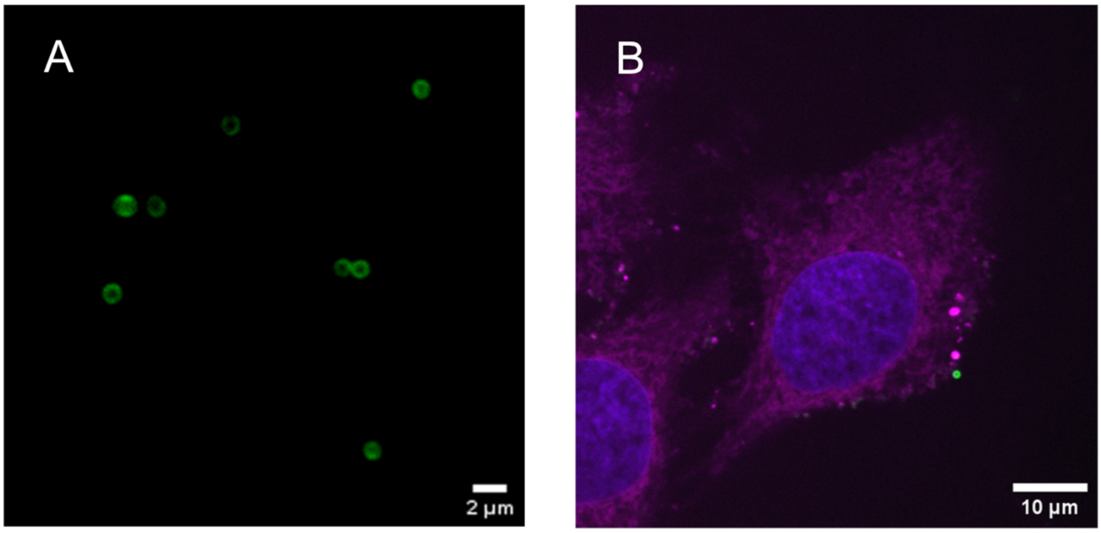

Figure S9. Fluorescent images *S. epidermidis* labeled with Van-BODIPY; (A) *S. epidermidis* was treated with 2 nM of Van-BODIPY, (B) *S. epidermidis* co-cultured with HeLa cell were treated with 1.5 µM Hoechst (nuclei = blue), 1 µM ER-Tracker™ Red (ER = magenta) and 10 nM Van-BODIPY (*S. epidermidis* = green).

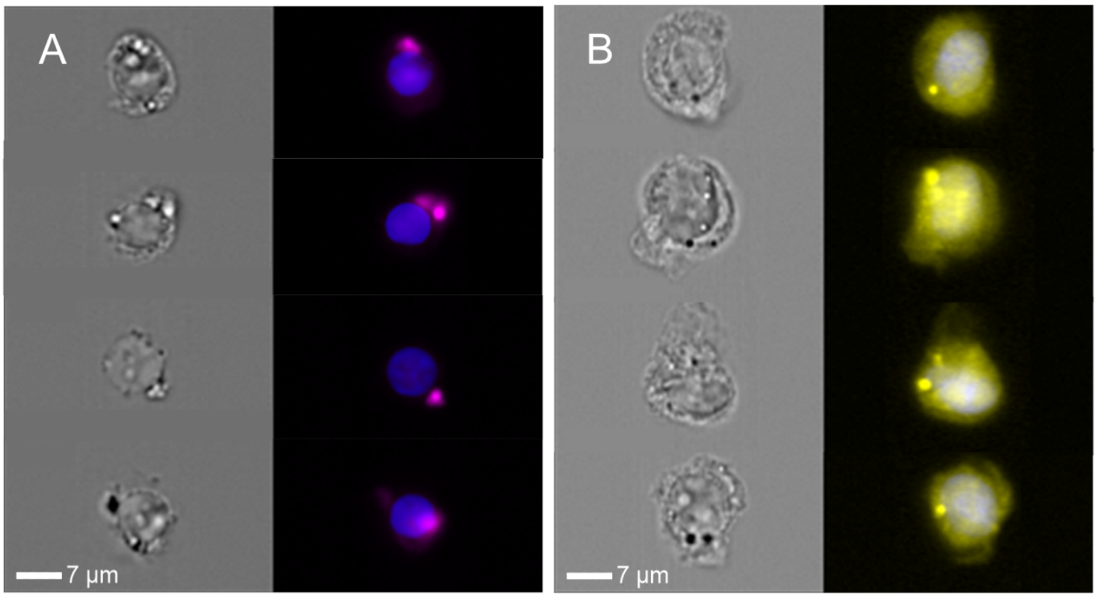

Figure S10. Further examples of flow cytometry images of in vitro co-culture of THP1 cells + clinical *S. aureus* (PROSA25) labeled by (A) 1.2 µM Van-JF_{669} and (B) 4.84 µM RADA treated with 3 µM DAPI (nuclei = blue).

4 Synthetic procedures

Scheme S1. Synthesis of vancomycin containing JF_{669} (Van-JF_{669})

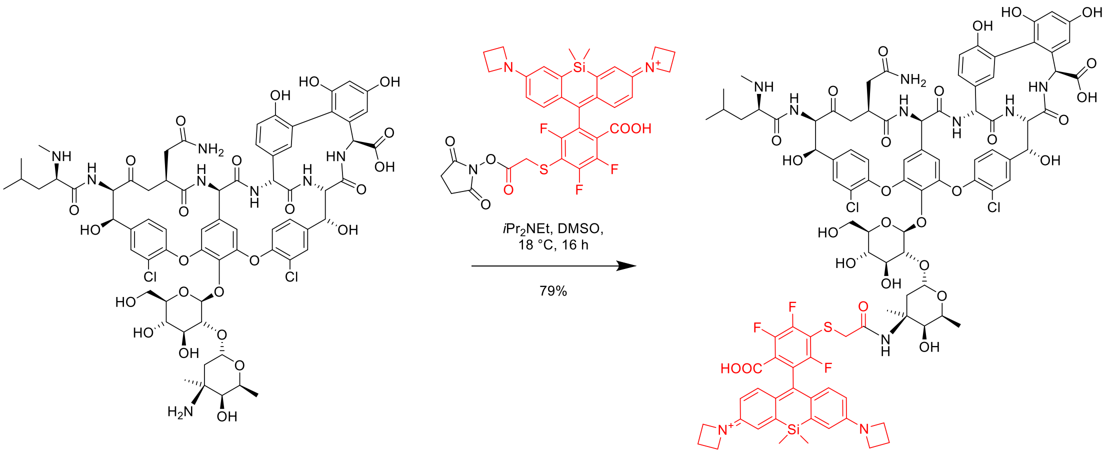

1-(10-(3-((2-(((2S,3S,4S,6S)-6-(((2S,3R,4S,5S,6R)-2-(((3S,6R,7R,22R,23S,26S,36R,38aR)-3-(2-amino-2-oxoethyl)-26-carboxy-10,19-dichloro-7,22,28,30,32-pentahydroxy-6-((R)-4-methyl-2-(methylamino)pentanamido)-2,5,24,38,39-pentaoxo-2,3,4,5,6,7,23,24,25,26,36,37,38,38a-tetradecahydro-1H,22H-23,36-(epiminomethano)-8,11:18,21-dietheno-13,16:31,35-di(metheno)benzo[n][1]oxa[6,9]diazacyclohexadecino[4,5-d][1]oxa[7,17]diazacyclotetracosin-44-yl)oxy)-4,5-dihydroxy-6-(hydroxymethyl)tetrahydro-2H-pyran-3-yl)oxy)-3-hydroxy-2,4-dimethyltetrahydro-2H-pyran-4-yl)amino)-2-oxoethyl)thio)-6-carboxy-2,4,5-trifluorophenyl)-7-(azetidin-1-yl)-5,5-dimethyldibenzo[b,e]silin-3(5H)-ylidene)azetidin-1-ium (**Van-JF_{669}**)

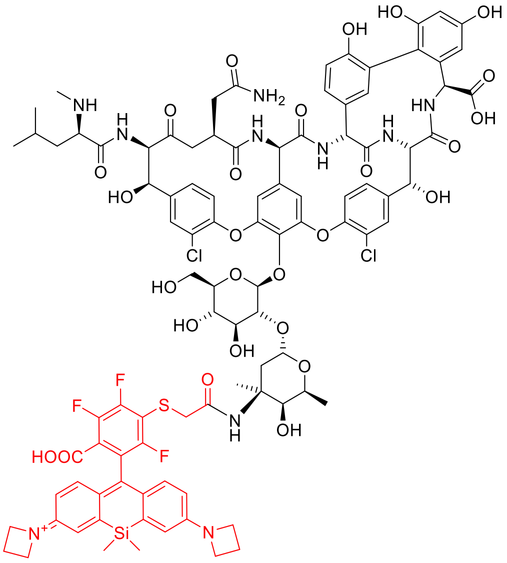

Vancomycin hydrochloride (1 mg, 0.67 µmol,), JaneliaFluor 669 NHS ester (700 µg, 1 µmol) and diisopropylethylamine (1 µL, 6 µmol) were stirred in dimethyl sulfoxide (500 µL) at room temperature for 16 hours. After that, the reaction was lyophilized. Next, acetonitrile (1 mL) and water (1 mL) were added and then purified by semi-preparative HPLC (elution gradient from acetonitrile/water = 1:9 to acetonitrile/water = 7:3) to give a blue powder (1 mg, 79%)

HRMS (ESI) $[M+H]^{2+}$ calculated for $[C_{96}H_{101}Cl_2F_3N_{11}O_{27}SSi]^{+}$: 1014.2894, found 1014.2879

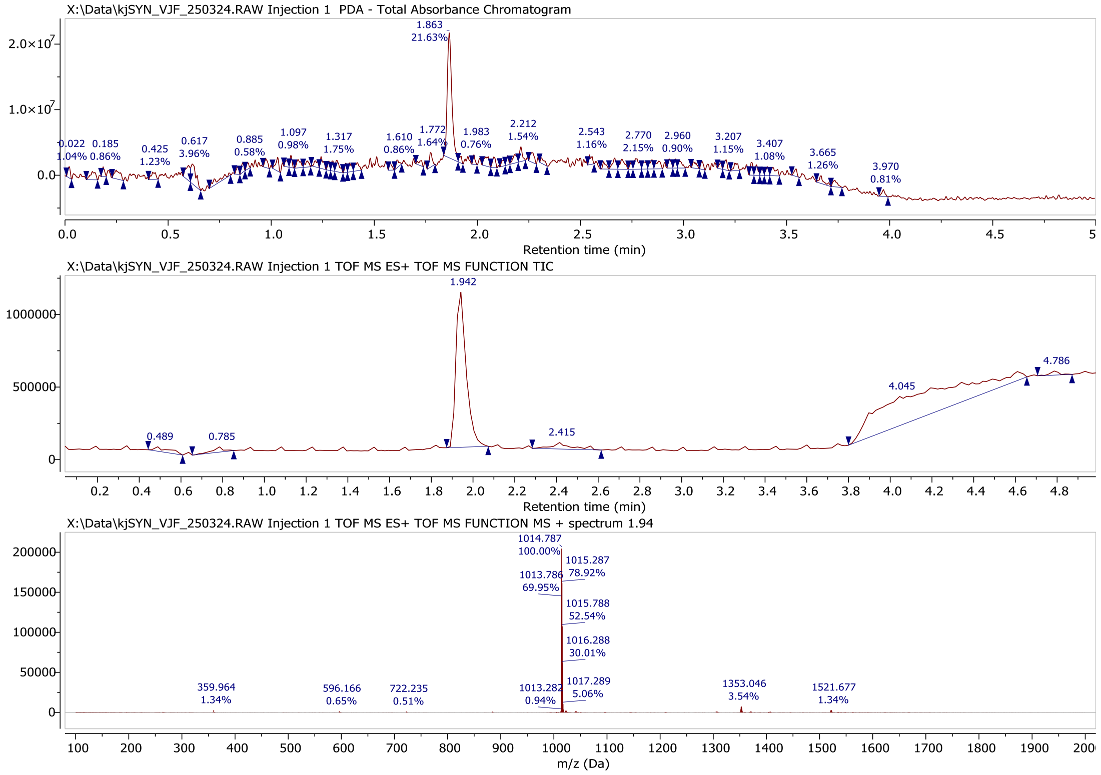

Figure S11. Integrated absorbance signal (100-300 nm) of analytical LC-MS run (10 → 95% acetonitrile in ddH_2O + 0.02% TFA + 0.04% FA over 3 min, then additional 2 min at 95%) of Van-JF_{669}.

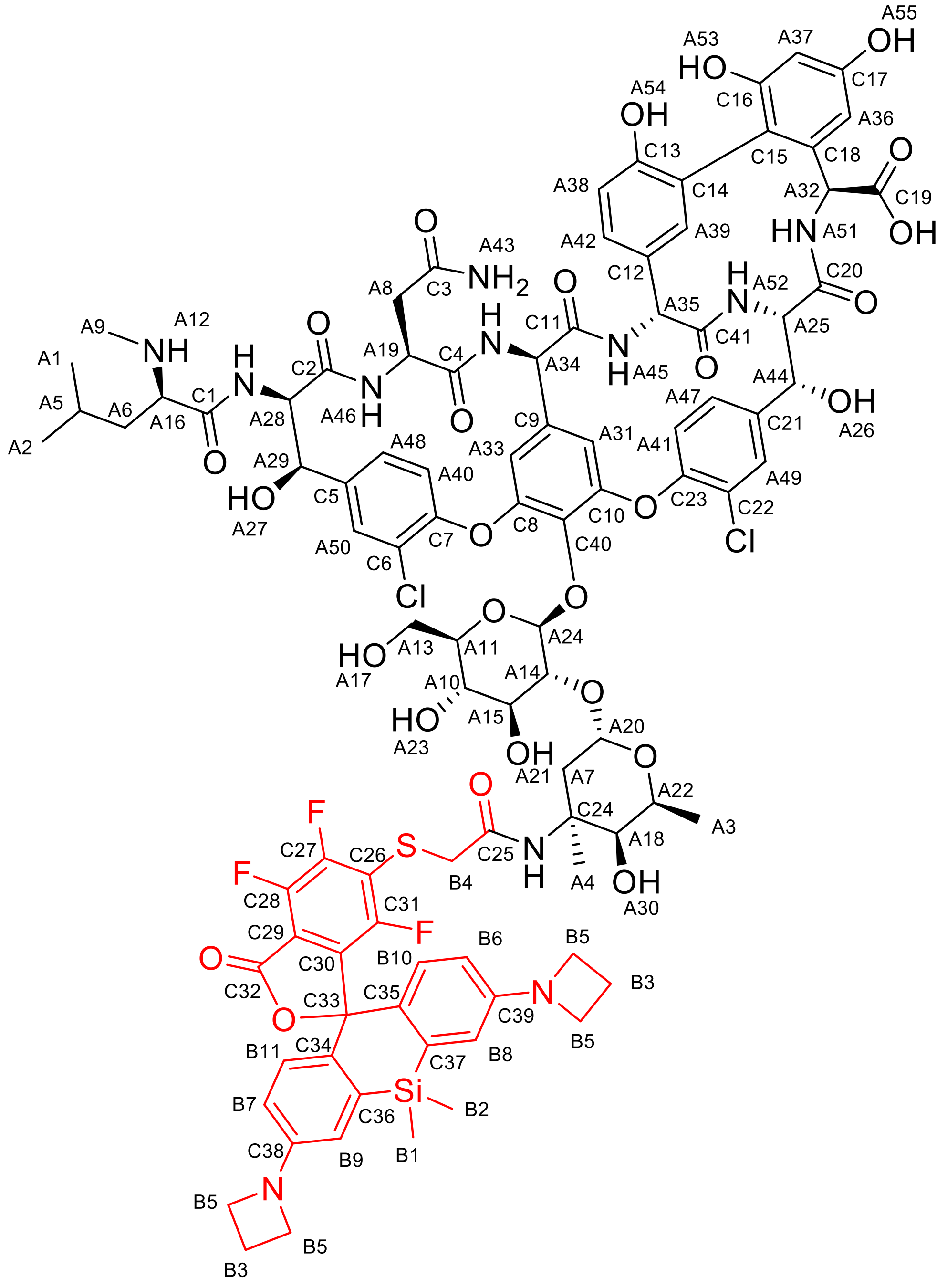

Figure S12. Labelled Van-JF_{669} for NMR elucidation

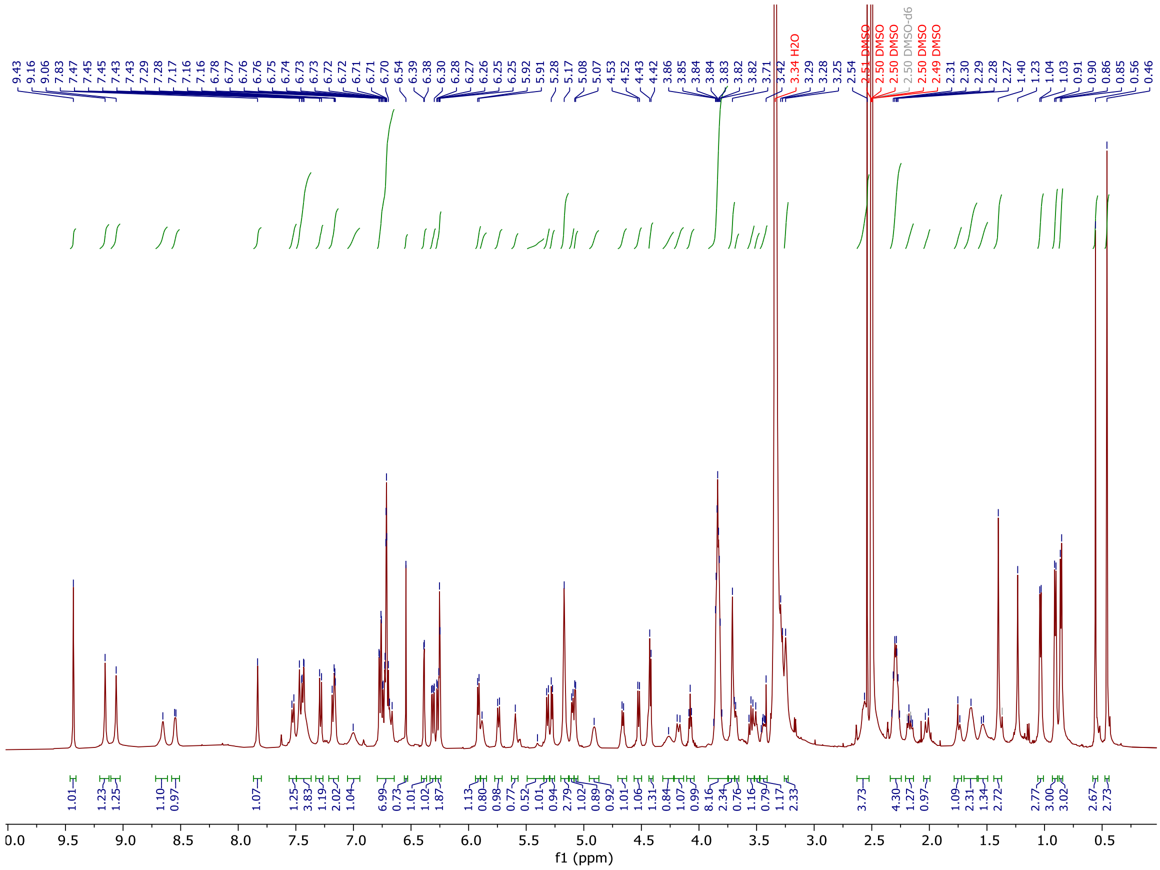

Figure S13. ^{1}H NMR of Van-JF$_{669}$

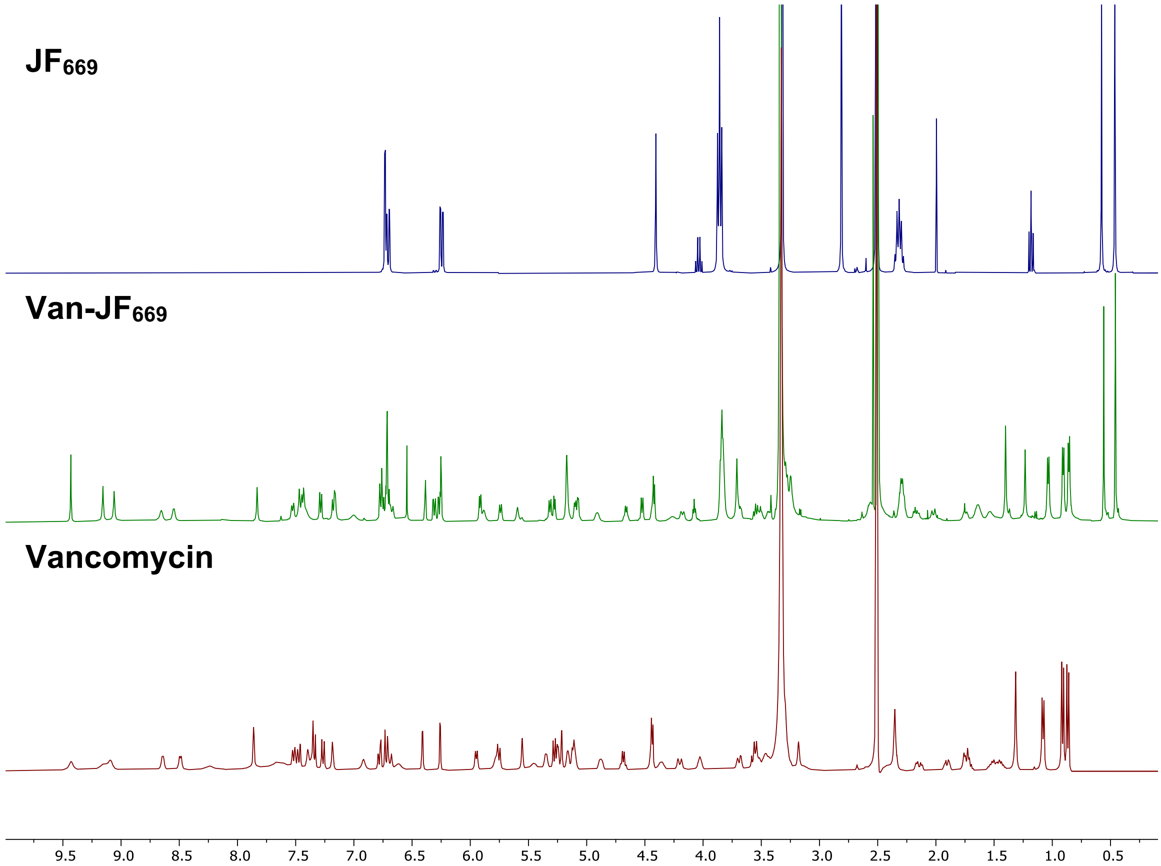

Figure S14. The overlay ^{1}H NMR of Vancomycin, Van-JF$_{669}$ and JF$_{669}$

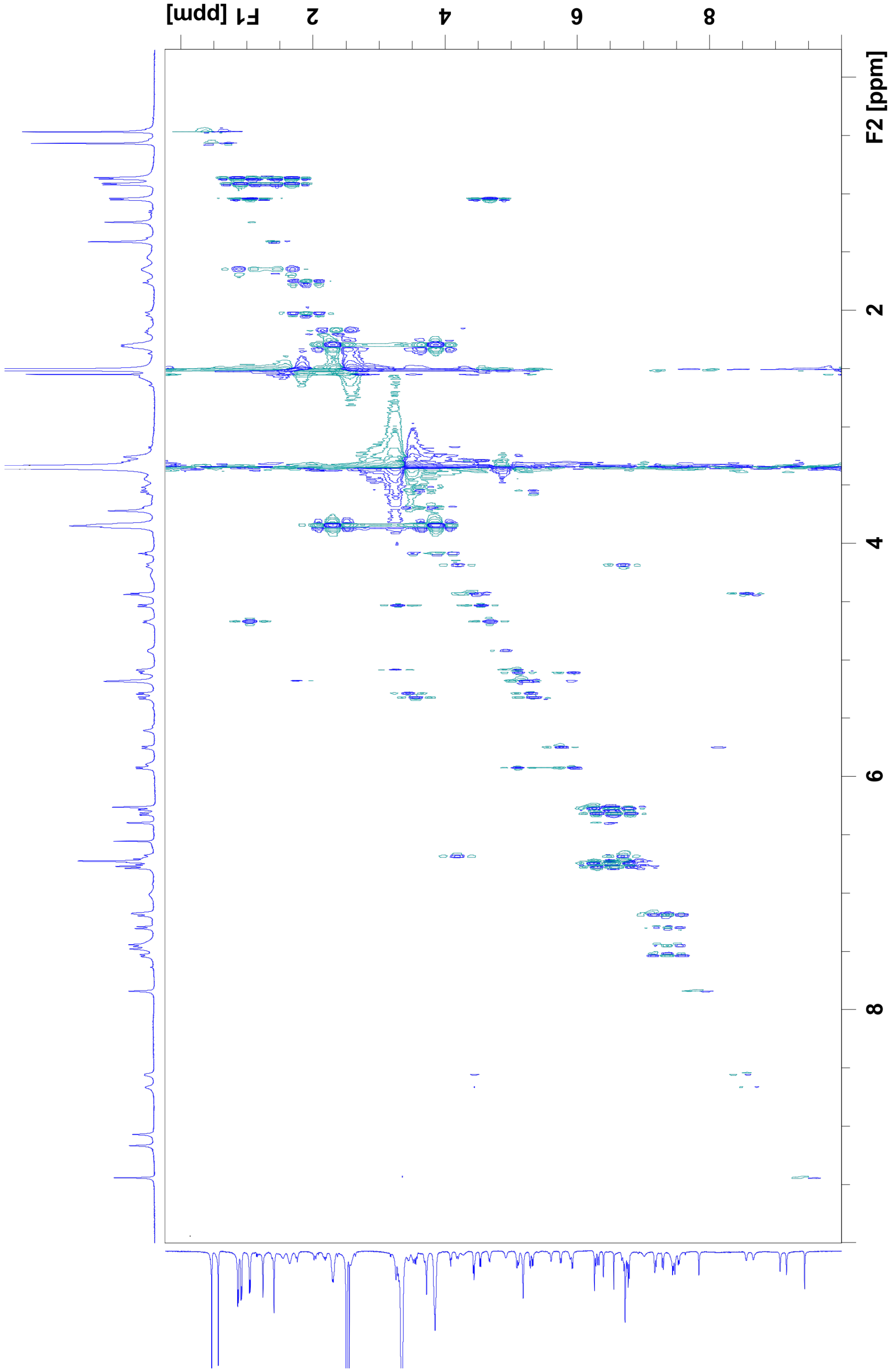

Figure S15. COSY spectra of Van-JF$_{669}$

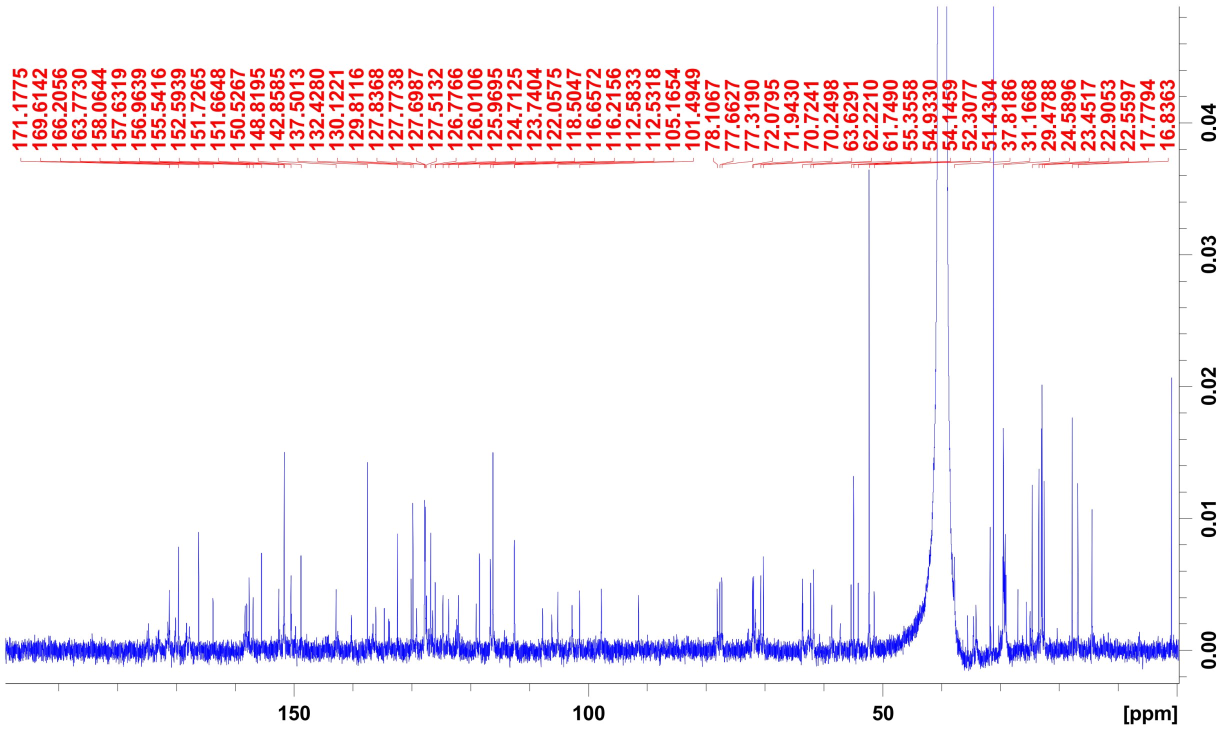

Figure S16. ^{13}C NMR of Van-JF$_{669}$

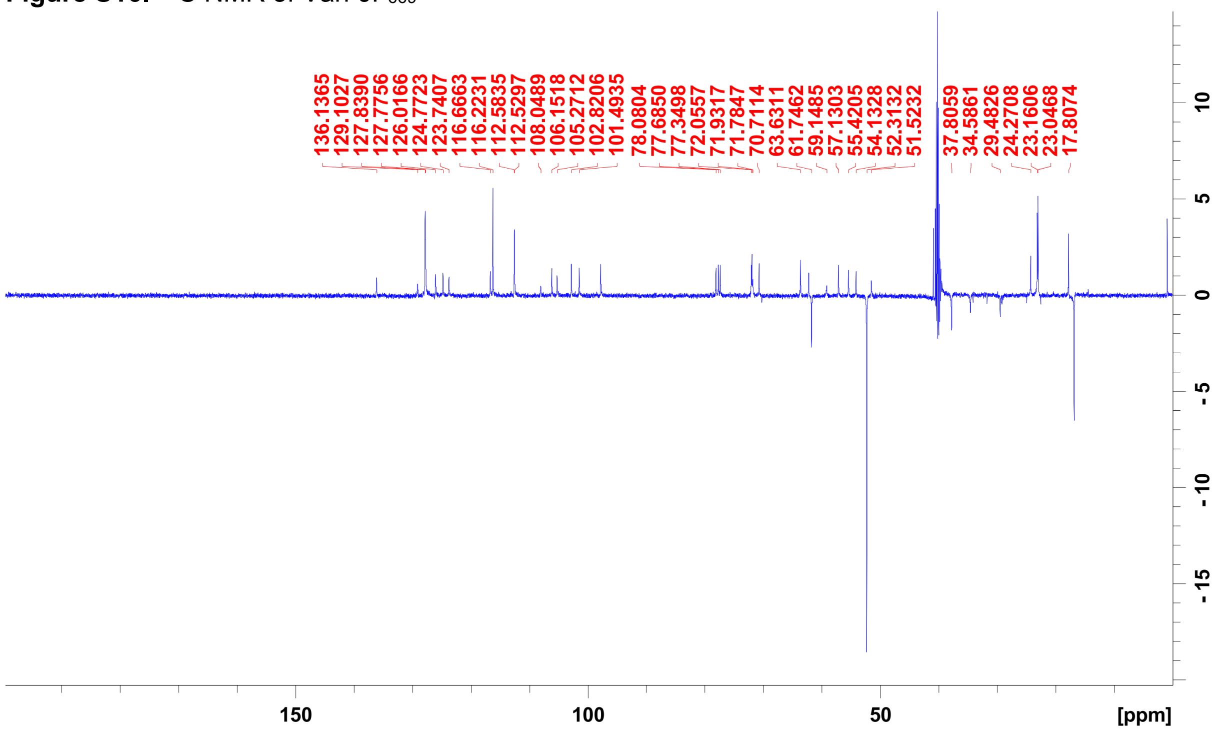

Figure S17. ^{13}C NMR (DEPT135) of Van-JF$_{669}$

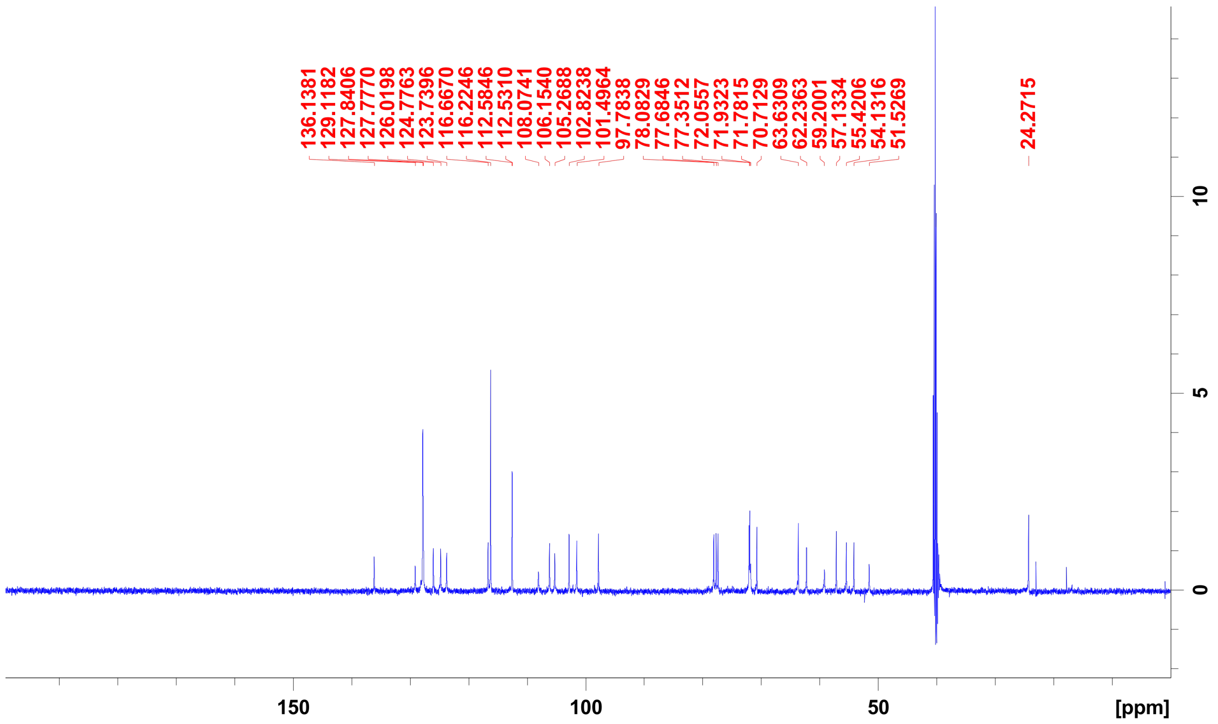

Figure S18. ^{13}C NMR (DEPT90) of Van-JF_{669}

Table S2. NMR data of Van-JF$_{669}$

Position	^{1}H	^{13}C	Position	^{1}H	^{13}C	Position	^{1}H	^{13}C
A1	0.86 (d, 6.1)	22.6	A55	9.43 (s)		C19		166.2
A2	0.90 (d, 6.1)	22.9	B1	0.46 (s)	0.9	C20		174.8
A3	1.03 (d, 6.3)	17.8	B2	0.56 (s)	0.9	C21		134.7
A4	1.40 (s)	23.4	B3	2.29 (m)	16.8	C22		129.8
A5	1.54 (m)	23.6	B4	3.51, 3.67	34.6	C23		155.5
A6	1.64 (m)		B5	3.84 (m)	52.3	C24		57.2
A7	1.74 (eq, d, 8.6), 2.02 (ax, d, 13.0)	34.1	B6	6.26 (dd)	112.6	C25		168.2
A8	2.17 (m), 2.56 (m)	29.5	B7	6.30 (dd)	116.2	C26		119.0
A9	2.54 (s)	31.2	B8	6.71	122.0	C27		150.5
A10	3.26	70.2	B9	6.73	123.7	C28		148.8
A11	3.26	77.6	B10	7.43 (d, 1.8)	129.1	C29		118.5
A12	3.28 (d, 7.8)		B11	7.45 (d, 1.8)	129.1	C30		129.1
A13	3.7 (s)	61.7	C1		170.1	C31		157.6
A14	3.44 (m)	78.1	C2		171.1	C32		163.8
A15	3.55 (t, 8.6)	77.3	C3		174.8	C33		78.1
A16	3.83 (m)	58.6	C4		171.4	C34		137.5
A17	4.08 (t, 5.5)		C5		133.9	C35		137.5
A18	4.18 (d, 11.7)	70.7	C6		129.2	C36		132.4
A19	4.26 (br s)	51.4	C7		152.6	C37		133.8
A20	4.43 (d, 6.3)	101.6	C8		142.8	C38		151.6
A21	4.53 (d, 7.2)		C9		127.8	C39		151.7
A22	4.67 (q, 6.6)	77.6	C10		142.8	C40		140.2
A23	4.91 (br s)		C11		171.4	C41		169.6
A24	5.07 (d, 4.5)	105.1	C12		130.1			
A25	5.10 (d, 6.1)	63.6	C13		157.0			
A26	5.17 (s)		C14		127.7			
A27	5.17 (s)		C15		119.0			
A28	5.28 (d, 5.5)	62.2	C16		158.3			
A29	5.40 (br s)	72.0	C17		158.0			
A30	5.31 (d, 7.6)		C18		136.6			
A31	5.59 (s)	107.7	A43	7.00 (br s)				
A32	5.74 (d, 7.8)	54.1	A44	7.16 (d)	71.9			
A33	5.88 (s)	112.5	A45	6.72 (d)				
A34	5.92 (d, 5.8)	55.3	A46	7.28 (d, 8.7)				
A35	6.54 (s)	54.9	A47	7.47 (s)	126.0			
A36	6.25 (d, 2.3)	106.2	A48	7.47 (s)	126.4			
A37	6.39 (d, 2.3)	102.8	A49	7.53 (d, 8.7)	126.8			
A38	6.67 (d, 11.4)	116.7	A50	7.83 (s)	127.5			
A39	7.18 (s)	136.1	A51	8.55 (br d, 5.6)				
A40	6.73 (d)	124.7	A52	8.65 (br s)				
A41	6.76 (d)	126.0	A53	9.06 (s)				
A42	6.78 (d)	127.8	A54	9.16 (s)				

References

(1) De Jong, N. W. M.; Van Der Horst, T.; Van Strijp, J. A. G.; Nijland, R. Fluorescent Reporters for Markerless Genomic Integration in Staphylococcus Aureus. *Sci. Rep.* **2017**, *7* (1), 43889. https://doi.org/10.1038/srep43889.

(2) Burian, M.; Rautenberg, M.; Kohler, T.; Fritz, M.; Krismer, B.; Unger, C.; Hoffmann, W. H.; Peschel, A.; Wolz, C.; Goerke, C. Temporal Expression of Adhesion Factors and Activity of Global Regulators during Establishment of Staphylococcus Aureus Nasal Colonization. *J. Infect. Dis.* **2010**, *201* (9), 1414–1421. https://doi.org/10.1086/651619.

(3) Schnitzbauer, J.; Strauss, M. T.; Schlichthaerle, T.; Schueder, F.; Jungmann, R. Super-Resolution Microscopy with DNA-PAINT. *Nat. Protoc.* **2017**, *12* (6), 1198–1228. https://doi.org/10.1038/nprot.2017.024.

(4) Martin, A.; Rivera-Fuentes, P. A General Strategy to Develop Fluorogenic Polymethine Dyes for Bioimaging. *Nat. Chem.* **2024**, *16* (1), 28–35. https://doi.org/10.1038/s41557-023-01367-y.

(5) Grimm, J. B.; Brown, T. A.; Tkachuk, A. N.; Lavis, L. D. General Synthetic Method for Si-Fluoresceins and Si-Rhodamines. *ACS Cent. Sci.* **2017**, *3*, 975–985. https://doi.org/10.1021/acscentsci.7b00247.
